# Outcome of Very Late Relapse in Patients with Hodgkin's Lymphomas

**DOI:** 10.1155/2011/707542

**Published:** 2010-12-29

**Authors:** Francesco Gaudio, Annamaria Giordano, Vincenzo Pavone, Tommasina Perrone, Paola Curci, Domenico Pastore, Mario Delia, Clara de' Risi, Alessandro Spina, Vincenzo Liso, Giorgina Specchia

**Affiliations:** ^1^Hematology Department, DAP, University of Bari, Piazza G.Cesare 11, 70124 Bari, Italy; ^2^Hematology Department, Cardinal G. Panico Hospital, Tricase, 73039 LE, Italy

## Abstract

Recurrences of Hodgkin's Lymphoma (HL) 5 years after the initial therapy are rare. The aim of this study is to report a single centre experience of the clinical characteristics, outcome, and toxicity of pts who experienced very late relapses, defined as relapses that occurred 5 or more years after the achievement of first complete remission. Of 532 consecutive pts with classical HL treated at our Institute from 1985 to 1999, 452 pts (85%) achieved a complete remission. Relapse occurred in 151 pts: 135 (29.8%) within 5 years and 16 over 5 years (3.5%, very late relapses). Very late relapses occurred after a median disease-free interval of 7 years (range: 5–18). Salvage treatment induced complete remission in 14 pts (87.5%). At a median of 4 years after therapy for very late relapse, 10 pts (63%) are still alive and free of disease and 6 (37%) died (1 from progressive HL, 1 from cardiac disease, 1 from thromboembolic disease, 1 from HCV reactivation, and 2 from bacterial infection). The probability of failure-free survival at 5 years was 75%. The majority of deaths are due to treatment-related complications. Therapy regimens for very late relapse HL are warranted to minimize complications.

## 1. Introduction

With MOPP/ABVD or ABVD regimens more than 70% of patients with Hodgkin's lymphoma (HL) can effectively be cured. Fewer than 30% of patients, particularly in advanced stage, may relapse after first-line treatment [1–3]. 

Several clinical and laboratory features have been used to predict progression-free survival (PFS) and overall survival (OS), in order to adjust therapy according to the risk of relapse. These features include age, sex, bulky disease, Ann Arbor stage IV, bone marrow involvement, anaemia, high serum levels of lactate dehydrogenase (LDH) or beta2-microglobulin [4–8], serum interleukin 10, and soluble CD30 [9–12].

Relapses usually occur within the first 3 years and a second complete remission can be achieved using treatment tailored according to disease extent at relapse [7] and previous treatment [13–16]. Few patients with documented very late relapses have been closely analyzed in the literature [17–31].

The aim of this study was to describe the incidence, clinical presentation, treatment, and outcome of very late relapse patients (defined as relapse occurring more than 5 years after complete remission) in a series of 532 consecutive patients with classical HL treated in our institution.

## 2. Patients and Methods

From 1985 to 1999, 532 consecutive previously untreated patients with classical HL were evaluated and treated at the Hematology Department of the University of Bari, Italy.

All patients were staged according to the Ann Arbor staging system. Initial evaluation included a complete medical history and physical examination, blood cell count and serum biochemistry profile, thoracoabdominal computed tomography (CT), and bone marrow biopsy at diagnosis and at relapse.

Response to treatment was defined according to the International Working Group recommendations [32]. 

Patients in complete remission after first-line chemotherapy were included in this study; a followup was carried out with clinical examination, blood counts, biochemical tests, chest X-ray or thoracic CT, and abdominal CT or ultrasound, performed every 3 months during the first 2 years after treatment completion, every 6 months during the following 3 years, and annually thereafter, with a median followup of 12 years.

In all patients who relapsed, a second biopsy was performed to prove the HL histology of relapse.

## 3. Statistical Analysis

STROBE reporting guidelines for observational studies were followed [33].

OS and FFS were estimated according to the Kaplan Meier product limit method.

Overall survival (OS) was measured from the date of the very late relapse to the last visit or death. Failure-free survival (FFS) was calculated from the date of very late relapse until documented relapse from CR after salvage therapy, disease progression following incomplete response to salvage therapy, or death, whatever came first. Deaths from unrelated causes were censored.

## 4. Results

After first-line therapy, 452 patients (85%) achieved complete remission (CR). 135 (29.8%) relapsed within 5 years (102 patients relapsed <3 years and 33 between 3 and 5 years) and 16 (3.5%) had very late relapse (>5 years).

The distribution of patients and relapse characteristics as a whole, and divided according to type of relapse, are shown in Table 1.

The incidence rate of very late relapse after 5-year disease-free interval was 3.5%.

The characteristics and the treatments of the patients in very late relapse are reported in Table 2.

### 4.1. Baseline Characteristics of Patients with Very Late Relapses

The histologies of the 16 patients in very late relapses were mixed cellularity in 7 (44%) and nodular sclerosis in 9 (56%). The median age was 37 yrs (16–70); 10 patients were female and 6 were male.

Ten patients (62%%) had stage I-II. B symptoms were present in 12 patients (75%), and 7 (44%) had bulky disease. First-line treatment was either standard or according to investigational protocols active during the time the patient was diagnosed. The chemotherapy schedules used at diagnosis were: ABVD in 1 (6%), MOPP/ABVD in 8 (50%), and MOPP in 6 (38%). Two patients in partial remission after MOPP/ABVD underwent high-dose chemotherapy (BEAM) and autologous stem cell transplantation. Involved-field radiotherapy was performed in 11 (69%) patients. The median radiation dose was 30 Gy (range: 20–36). One patient was treated with radiotherapy alone at diagnosis.

### 4.2. Patients Characteristics at Relapse

Median time to late relapse was 7 years; very late relapses occurred 5 to 18 years after treatment start (Table 2).

The histologies of very late relapses were in all cases the same as at diagnosis. 

Clinical presentation at relapse demonstrated a supradiaphragmatic localization in 6 cases (37%), infradiaphragmatic in 8 (50%) and located on both sides in 2 cases (13%). 

Bulky nodal disease was observed in 4 patients (25%). Extranodal disease occurred in 1 patient (6%). B symptoms were present in 6 patients (37%).

One patient (6%) relapsed on the irradiated field (mediastinal); 5 (31%) relapsed in sites of bulky disease; in 6 patients (37%), the disease localization was different from the site at diagnosis.

### 4.3. Treatments of Very Late Relapse and Followup

Treatment of relapse consisted of chemotherapy alone in 13 pts (81%) and chemotherapy and radiotherapy in 3 (19%). Among 6 patients initially treated with MOPP/ABVD, 4 were retreated with MOPP/ABVD and 2 received ABVD. All 6 patients initially treated with MOPP received noncross-resistant chemotherapy, 5 with ABVD and 1 with MOPP/ABVD. Both patients who relapsed following MOPP/ABVD plus BEAM + ASCT received escalated BEACOPP. The only patient who relapsed after RT alone received VEPEM-B. Only 1 patient received HDT/ASCT as second-line treatment for very late relapse.

In 4 patients, the chemotherapy regimen was the same one used as front-line therapy, and all patients achieved a second complete remission.

6/16 patients failed salvage therapy (2 PR and 4 relapses), and 2 additional patients died in second CR due to thromboembolic disease and HCV infection, 2 and 29 months after the confirmation of second CR, respectively.

Of the 16 patients, 14 achieved a second complete remission (87%) and 9 patients are still alive and free of disease. Two patients achieved a partial remission.

The median followup was at 32 months (range: 8–250), 6 patients died (Table 2).

Four patients experienced a second relapse within 1 year from the second complete remission, and they relapsed in the same sites as at the first relapse. One of them was reinduced to CR with the IEV regimen and is still in CR with a followup of 24 months, the other three patients died from toxicity during chemotherapy salvage treatment (2 from Gram-negative septic shock, 1 of cardiotoxicity). The patient who died of cardiac failure had a cumulative dose of anthracycline of 300 mg/sqm (the median cumulative dose of anthracycline for all patients was 300 mg/sqm). 

In addition, one patient died from thromboembolic disease, and one died from HCV reactivation without evidence of HL (Table 2).

Of the two patients in PR, one achieved a CR with the IEV regimen and is still in CR after 14 months of followup while the other one died of HL progression.

**Figure 1:**
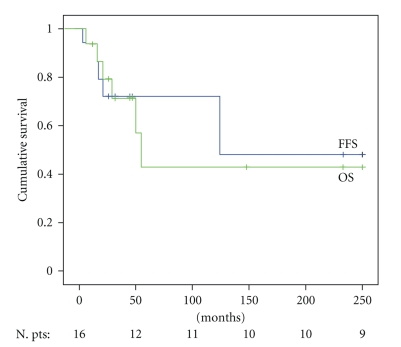
Overall survival and failure-free survival in 16 PTS with VLR HL. PTS: patients; VLR: very late relapses; FFS: failure-free survival; OS: overall survival.

The estimated OS after late recurrence was 44% at 5 years. The estimated FFS was 75% at 5 years (Figure 1).

## 5. Discussion

The majority of patients with HL can be cured with conventional chemotherapy (ABVD), radiotherapy, or both. Even though HL is curable, approximately 30% of all patients relapse and eventually die of disease progression or complications of therapy. [1–3]. Most studies focused on early relapses should lead to improved first-line treatment approaches and high survival rates. Much less is known about very late relapse in terms of incidence and outcome [17–31].

There is no consensus on the definition of very late relapse: three or five years have been proposed in various reports [17–27]. An interval of 5 years was chosen because the actuarial rate of relapse appeared to reach a plateau at 5 years [17–27].

The incidence of late relapse reported in previous studies was variable, from 3.5 to 8%. This difference is probably due to a different definition of the time of relapse (three, five years, or more) [17–31].

As the incidence of very late relapse in HL is higher than the incidence of HL in the general population, it suggests that this event represents a reactivation of the disease (in the same sites and with the same histology), rather than the development of a new HL [22]. The finding of persistent HL in autopsies of long survivors who died of apparently unrelated causes suggests that, for at least some patients with HL, clinical cure does not always mean the eradication of all the malignant cells [34]. Some authors have suggested that the circumstances that impaired the immune system may cause the recurrence [35]. At least part of the late recurrences of HL might be de novo emerging malignancies in patients at elevated genetic risk for developing HL [36]. The persistence of the same viral strain in early and very late relapse of Epstein-Barr virus is evidence that in HL such relapses are related to a single residual tumor cell clone [37, 38].

Herman et al. [25] reported that the actuarial risk of relapse after a 3-year disease-free interval was 13%. These investigators found that the occurrence of late relapse was significantly related to stage I disease and the nodular sclerosis histologic subtype. Another report, by the European Organization for Research and Treatment of Cancer [22], showed a 3.5% incidence of very late relapse after a 5-year disease-free interval. That study showed that the incidence of late relapses was significantly correlated with male sex, B symptoms, mediastinal involvement, and treatment modality. The authors found that HL patients who experience a very late relapse have a similar survival to patients who are continuously disease-free. The actuarial incidence of very late relapse at 10 and 15 years in early stage patients was 4.8% and 8.3%, respectively. Similarly, the actuarial incidence of very late relapse at 10 and 15 years in patients of all stages was 4.4% and 8.0%, respectively, as reported by Vassilakopoulos et al. [27].

The investigators observed that patients with LH more often developed very late relapse when they were treated with less intensive therapy. In our series, two patients had very late relapse after high-dose chemotherapy and autologous stem cell transplantation. Another report [20] of patients in early stage HL revealed 4.6% of very late relapse (more than 4 years). These investigators found that these patients had better survival than did patients with early relapse.

Bodis et al. [22] reported that very late relapses in early stage patients were more frequent in males with B symptoms at diagnosis and mediastinal involvement, who had been treated with radiotherapy only (versus combined modality). Vassilakopoulos et al. [27], in an analysis of all stages, reported that very late relapses were more frequent in patients with more extensive disease at diagnosis, nonnodular sclerosing histology, who had been treated with chemotherapy only (versus combined modality). Also Brierly et al. [20] found age and B symptoms as predictors for late relapse in early stage patients.

The fact that in our study, 14 patients (87%) had stage I or II disease at the time of very late relapse could be due to the continuous followup of patients in complete remission.

No histological characteristics have been found to correlate with very late relapse risk, but more cases are necessary to draw definitive conclusions. There is no accurate means to identify patients at risk of very late relapse, and there is no absolutely safe point at which an individual patient may be considered cured of HL and at no risk of very late relapse [17–31].

In our study 11/16 patient had radiotherapy as part of their initial therapy, but only one patient relapsed in the radiotherapy field. Radiotherapy seems to be an important issue in preventing late relapse.

Patients who relapse within the first 3 years have more aggressive disease, and patients who relapse late have a more indolent disease that responds to further therapy [21, 22]. Very late relapse is adequately reinduced to complete remission by a second course of the primary treatment regimen, and so are still curable with conventional treatment, as documented by our 5 patients treated with the same therapy, who achieved a second complete remission. In fact, 5/6 patients treated with the same regimen (4 MOPP/ABVD to MOPP/ABVD, 2 MOPP/ABVD to ABVD) achieved a second CR, but 2/5 relapsed, and one is not evaluable for relapse due to early death. Thus the data presented in this study support that the administration of the same drugs is effective in inducing a second CR, but its potential in inducing long-term remissions (cure?) is questionable.

However, despite the high percentage of complete remissions (87%), 4 patients relapsed (second relapse) early (<1 years) while 4 patients died of toxicity (1 from cardiac disease, 1 from HCV reactivation, and 2 from gram negative sepsis). Another TRM due to pulmonary thromboembolism was observed in second complete remission, 2 months after the completion of chemotherapy.

In conclusion, very late relapse occurs in a small number of patients with HL, which emphasizes the need for continuous followup. Very late relapse is associated with a better survival than relapse occurring within the first 5 years from the time of diagnosis [20, 22]. Unfortunately, the majority of deaths of patients in very late relapse are related to treatment complications while deaths due to HL are unusual. Future treatment regimens for HL, including very late relapse, need to be designed to minimize complications.

## Figures and Tables

**Table 1:** Patients characteristics of HL in complete remission after first-line chemotherapy.

	All	Early relapse	Late relapse
*n*	452	135	16
Median age (range)	31 (14–86)	36 (16–79)	37 (16–70)
Male/Female	244/208	81/54	6/10
Stage I-II	271 (60%)	52 (39%)	10 (62%)
Stage III-IV	181 (40%)	83 (61%)	7 (44%)
B symptoms	158 (35%)	58 (43%)	12 (75%)

Histology:			
NS	275 (61%)	73 (54%)	9 (56%)
MC	168 (37%)	58 (43%)	7 (44%)
LD	9 (2%)	4 (3%)	0 (0%)

NS: nodular sclerosis; MC: mixed cellularity; LD: Lymphocyte depletion.

**Table 2:** Patients characteristics of HL with very late relapse.

	Time to relapse	AGE at DG	Sex	Histol.	First-Line regimen	Lymphoma localization		Therapy	Responce	Second relapse	Cause of death	OS	FFS
						at diagnosis	at relapse	at relapse				mths	mths
1	5	46	F	NS	MOPP/ABVD	INFRA	SUPRA	MOPP/ABVD	CR	YES	GRAM- sepsis	55	17
2	5	23	F	MC	MOPP/ABVD	INFRA	SUPRA	MOPP/ABVD	CR	NO		250	250
3	5	70	M	NS	MOPP + I.F.Radioth.	SUPRA	SUPRA	MOPP/ABVD	PR		Disease progr.	16	16
4	8	20	F	NS	HDS-ASCT + I.F.Radioth.	INFRA + SUPRA	INFRA	BEACOPP	CR	NO		32	32
5	18	37	F	NS	MOPP + I.F.Radioth.	SUPRA	INFRA	ABVD	CR	NO		32	32
6	8	20	F	MC	MOPP/ABVD + I.F.Radioth.	SUPRA	INFRA + SUPRA	ABVD	CR	NO		233	233
7	5	16	F	NS	MOPP	INFRA + SUPRA	INFRA	ABVD	CR	YES		148	124
8	5	66	F	MC	MOPP + I.F.Radioth.	SUPRA	INFRA	ABVD	CR	NO		47	47
9	5	56	F	MC	MOPP/ABVD	SUPRA	INFRA	MOPP/ABVD	CR	NO	Tromboembolic D.	8	8
10	11	32	F	NS	MOPP + I.F.Radioth.	SUPRA	SUPRA	ABVD	CR	YES	GRAM- sepsis	21	21
11	10	22	F	MC	HDS-ASCT + I.F.Radioth.	INFRA + SUPRA	INFRA	BEACOPP	CR	NO		8	8
12	9	68	M	MC	I.F.Radioth.	SUPRA	INFRA	VEPEM-B	CR	NO		45	45
13	6	52	M	MC	MOPP	INFRA + SUPRA	SUPRA	ABVD	CR	NO	HCV reactivation	29	29
14	7	35	M	NS	MOPP/ABVD + I.F.Radioth.	INFRA + SUPRA	INFRA	MOPP/ABVD	CR	YES	cardiac failure	50	30
15	7	18	M	NS	MOPP/ABVD + I.F.Radioth.	INFRA + SUPRA	INFRA + SUPRA	ABVD	PR			26	26
16	5	18	M	NS	ABVD + I.F.Radioth.	SUPRA	SUPRA	IGEV-ASCT	CR	NO		12	12
